# Is Cancer Curable?

**Published:** 1875-12

**Authors:** E. H. Gibbs


### IS CANCER CURABLE?

BY E. H. GIBBS, M. D.

It should be known to everyone, that Cancerous Affections, located on the surface of the body, and within reach of local remedies, may generally be cured, if subjected to proper treatment before the disease has had time to contaminate the system. After that, the treatment is only palliative, but may serve to prolong life in comfort, for many years, and save the patient from those evidences of the disease which are most repulsive. No people and no country are exempt from this fearful malady, which, for more than two thousand years, has baffled the best medical skill.

Nearly all other terrible diseases have been conquered. This, the most malignant of all, has been the last to yield to medical science. Cancer, or Carcinoma, is a peculiar morbid growth, with the property of indefinite propogation; the power of contaminating and destroying all neighboring tissues; of poisoning the blood, and overthrowing life. Cancer may lie dormant for years, and the individual may go to his grave at a good old age, through some other disease; but when once aroused to action, the march of this terrible disease is towards death, unless promptly arrested and totally destroyed.

It first appears in a small spot or in several spots, nearly contiguous, which coalesce and extend by invading and feeding upon the healthy tissues, forming a tumor which varies in shape and size according to circumstances.

The tumor appears to have a vascular connexion with the surrounding parts, but the veins are filled with cancerous matter, so that it is impossible to inject them; hence the varicose condition of the superficial blood-vessels. Every organ, and almost every tissue of the body is liable to become the seat of cancer. It is usually attended with sharp, lancinating pains, which increase as the ulceration extends. When the system becomes affected, there is often emaciation, weakness, disordered digestion, febrile symptoms, a wan, sallow paleness and an expression of sadness. The patient finally sinks under exhaustion from pain, or excessive discharges, or by the interference of the disease with the function of some organ essential to life. Even his brief description of the disease would not be given in this article, were it not to quiet the fears of those who are suspicious of every sore or swelling they can not account for. Many of the affections treated as cancers, by quacks, and usually made worse by them, are as harmless and inert as a simple wound or bruise. Between the abuses perpetrated by this class of men, and the mistaken views of many educated physicians, the unfortunate sufferers from cancer have been made the victims of unutterable misery since the world began. But the course of medical science is onward. The long night of ignorance is passing away. Medical men easily conquer diseases that were regarded as incurable, even ten years ago. The light that has been shed on the origin, the cause and the course of this most fearful of all maladies, plainly indicates what should be its treatment. Like all constitutional diseases, it has a germinal origin. Until recently, the best medical authorities believed cancer to be an hereditary taint of the system which, aroused to action from any sufficiently exciting cause, commenced its ravages in the circulation; and that its appearance on the surface, was only symptomatic of a constitutional trouble. But long and careful investigation, seems to have established THREE, all important facts: *First*, that cancer is not essentially an hereditary disease. *Second*, that the germ is located at the point where the ulceration begins. *Third*, that the contamination of the rest of the system does not commence until some time after the disease has made its appearance.

The inconvenience and danger to the patient is always increased, in proportion to the delay of the treatment. Where the eradication of the diseased part is prompt and thorough, the disease seldom returns. After an experience of the best part of a life-time in some of the leading hospitals in this country and in Europe, I unqualifiedly condemn the use of the knife, in extirpating cancer. In no case, of the hundreds observed by me, has the operation proved more than palliative. I could give satisfactory reasons for condemning its use in all cases, did the limits of this article permit. When cancer attacks an internal organ, it is beyond the reach of remedies and its fatal termination, is simply a question of time. If there is any compensation for its unfortunate location, it is in sparing the sufferer and his friends from a view of its most repulsive features.

Let me urge it as a sacred duty, incumbent on every rational human being, on the appearance of any suspicious sore or tumor, or other disease, to consult without delay an experienced physician. The general adoption of such a course would save a world of suffering, and thousands of lives. Those little words, "too late," recall the saddest recollections of a physician's life. My interest in this important subject is great, as must be that of all intelligent and studious physicians. I should be glad to correspond with my brother physicians upon this topic, and to compare notes of treatment, failure and success. I can be addressed at 137 Eight Street New York.

